# Supplementary material for: Resolving the full spectrum of human genome variation using Linked-Reads
Source: Genome Res. 2019 Apr;29(4):635–45. doi: 10.1101/gr.234443.118 (PMC6442396; doi:10.1101/gr.234443.118)
Supplement: Supplemental Material [file supp_gr.234443.118_Supplemental_longranger-2.2.zip › longranger-2.2.tar.bz2!/lr2.2-public/lib/stan/cmdstan/stan/doc/api/html/math_2error__handling_8hpp_source.html]

Stan: src/stan/math/error\_handling.hpp Source File


|  |  |
| --- | --- |
|  | Stan  2.5.0  probability, sampling & optimization |


- Main Page
- Related Pages
- Namespaces
- Classes
- Files

- File List
- File Members

All Classes Namespaces Files Functions Variables Typedefs Enumerations Enumerator Friends Macros Pages

- src
- stan
- math

error\_handling.hpp

Go to the documentation of this file.

1 #ifndef STAN\_\_MATH\_\_ERROR\_HANDLING\_HPP

2 #define STAN\_\_MATH\_\_ERROR\_HANDLING\_HPP

3

4 #include <algorithm>

5 #include <cstddef>

6 #include <limits>

7 #include <utility>

8 #include <iostream>

9

10 #include <boost/type\_traits/is\_unsigned.hpp>

11

12 #include <stan/meta/traits.hpp>

13 #include <stan/math/error\_handling/dom\_err.hpp>

14 #include <stan/math/error\_handling/dom\_err\_vec.hpp>

15 #include <stan/math/error\_handling/check\_not\_nan.hpp>

16 #include <stan/math/error\_handling/check\_equal.hpp>

17 #include <stan/math/error\_handling/check\_finite.hpp>

18 #include <stan/math/error\_handling/check\_greater.hpp>

19 #include <stan/math/error\_handling/check\_greater\_or\_equal.hpp>

20 #include <stan/math/error\_handling/check\_less.hpp>

21 #include <stan/math/error\_handling/check\_less\_or\_equal.hpp>

22 #include <stan/math/error\_handling/check\_bounded.hpp>

23 #include <stan/math/error\_handling/check\_nonnegative.hpp>

24 #include <stan/math/error\_handling/check\_positive.hpp>

25 #include <stan/math/error\_handling/check\_positive\_finite.hpp>

26 #include <stan/math/error\_handling/check\_consistent\_size.hpp>

27 #include <stan/math/error\_handling/check\_consistent\_sizes.hpp>

28

29 #endif

30

check\_not\_nan.hpp

dom\_err.hpp

check\_positive\_finite.hpp

check\_positive.hpp

check\_consistent\_size.hpp

traits.hpp

check\_greater.hpp

check\_nonnegative.hpp

check\_less\_or\_equal.hpp

check\_equal.hpp

check\_bounded.hpp

dom\_err\_vec.hpp

check\_finite.hpp

check\_greater\_or\_equal.hpp

check\_less.hpp

check\_consistent\_sizes.hpp


---

[ Stan Home Page ]

*© 2011–2014,
Stan Development Team.*
